# Mental Health Consequences of SARS-CoV-2 Pandemic on Long-Term Care Facility Social Care Provider in Poland

**DOI:** 10.1093/geroni/igab046.1576

**Published:** 2021-12-17

**Authors:** Dorota Szcześniak, Adrianna Senczyszyn, Maria Maćkowiak, Marta Ciułkowicz, Katarzyna Lion, Joanna Rymaszewska

**Affiliations:** 1 Department of Psychiatry, Wroclaw Medical University, Poland, Wroclaw Medical Univeristy, Dolnoslaskie, Poland; 2 Menzies Health Institute Queensland, Griffith University, Australia, Menzies Health Institute Queensland, Queensland, Australia

## Abstract

During the pandemic long-term care facilities (LTCF) social health providers constantly remain in a dilemma between loyalty to people with dementia and concerns for their own families. All of these factors could contribute to the mental burden, burnout, and increased chance of depression, anxiety and post-traumatic symptoms. In our study we aimed to provide a window on psychopathological consequences (somatic symptoms, anxiety and insomnia, social dysfunction, and depression) associated with the exposure of LTCF employees to the risk of the SARS-CoV-2 contagion in Poland. Moreover, we investigated if institutional factors, such as personal protection equipment availability, safety guidelines or access to psychiatric and psychological support at the workplace, contribute to the decrease of psychological distress of the LTCF personnel. The results can serve as ready-made guidelines for mitigating the SARS-CoV-2 impact on dementia care and constitute the basis for further analysis of long-term consequences of this precedential situation.

